# Maternal eating disorders affect offspring cord blood DNA methylation: a prospective study

**DOI:** 10.1186/s13148-017-0418-3

**Published:** 2017-10-27

**Authors:** Nabila Kazmi, Tom R. Gaunt, Caroline Relton, Nadia Micali

**Affiliations:** 1 MRC Integrative Epidemiology Unit, School of Social and Community Medicine, University of Bristol, Bristol, BS8 2BN UK; 2 Child and Adolescent Mental Health, Population Policy and Practice, Institute of Child Health, UCL, London, UK; 3 Department of Psychiatry, Icahn School of Medicine at Mount Sinai, New York, NY USA

**Keywords:** ALSPAC, Eating disorders, Methylation, Cord blood, Epigenetic

## Abstract

**Background:**

Eating disorders (ED) are chronic psychiatric disorders, common amongst women of reproductive age. ED in pregnancy are associated with poor nutrition and abnormal intrauterine growth. Increasing evidence also shows offspring of women with ED have adverse developmental and birth outcomes. We sought to carry out the first study investigating DNA methylation in offspring of women with ED. We compared cord blood DNA methylation in offspring of women with active ED (*n* = 21), past ED (*n* = 43) and age- and social class-matched controls (*n* = 126) as part of the Avon Longitudinal Study of Parents and Children.

**Results:**

Offspring of women with both active and past ED had lower whole-genome methylation compared to controls (active ED 49.1% (95% confidence intervals 50.5–47.7%), past ED 49.2% (95% CI 50.7–47.7.0%), controls 52.4% (95% CI 53.0%–51.0%)). Amongst offspring of ED women, those born to women with restrictive-type and purging-type ED had lower methylation levels compared to those of controls. Offspring of women with an active restrictive ED in pregnancy had lower whole-genome methylation compared to offspring of women with past restrictive ED. We observed decreased methylation at the *DHCR24* locus in offspring of women with active pregnancy ED (effect size (ES) = − 0.124, *p* = 6.94 × 10^−8^) and increased methylation at the *LGALS2* locus in offspring of women with past ED (ES = 0.07, *p* = 3.74 × 10^−7^) compared to controls.

**Conclusions:**

Maternal active and past ED are associated with differences in offspring whole-genome methylation. Our results show altered DNA methylation in loci relevant to metabolism; these might be biomarkers of disrupted metabolic pathways in offspring of ED mothers. Further work is needed to examine potential mechanisms and functional outcomes of the observed methylation patterns.

**Electronic supplementary material:**

The online version of this article (10.1186/s13148-017-0418-3) contains supplementary material, which is available to authorized users.

## Background

Eating disorders (ED; anorexia nervosa, bulimia nervosa, binge eating disorder, and other specified feeding and eating disorders) are severe psychiatric disorders that affect about 10% of women of reproductive age [1–3]. Maternal ED, both active—during pregnancy—and past, have been shown to affect child eating, growth, psychopathology and development [4–8]. However, little is known about biological risk mechanisms [9]. Women with active and lifetime ED differ from non-ED women in terms of their food intake during pregnancy, with differences highlighted in micronutrient and macronutrient intakes [10, 11]. Nutrient intake in pregnancy is affected by ED type, with differences in diet composition seen in women with anorexia nervosa (AN) compared to bulimia nervosa (BN) and binge eating disorder (BED) [10, 11].

Maternal food intake in utero, in terms of quality and quantity, is likely an important environmental determinant affecting foetal metabolic adaptation to the prevailing environmental conditions, as shown by studies on maternal nutritional deprivation as a result of widespread famine [12, 13]. Increasing evidence has highlighted the effect of maternal nutrition in pregnancy on epigenetic modifications, in particular DNA methylation, both in animals and humans [14, 15]. Low micronutrient (methyl donors) and protein intake, as well as low maternal pre-pregnancy BMI, have been prospectively associated with lower global methylation levels and hypomethylation at specific sites [14, 16].

No studies to date have investigated the effect of maternal ED on offspring cord blood methylation. Given that nutrition in pregnancy is likely to differ in women with ED compared to healthy women, our first aim was to carry out an epigenome-wide association study (EWAS) in offspring of mothers with ED and offspring of non-ED mothers. There is evidence that maternal nutrition might affect offspring methylation not just in utero but also preconception [15]; we therefore also aimed to disentangle the effect of active ED in pregnancy vs. ED at any point prior to pregnancy, by investigating both global methylation levels and methylation at CpG sites in offspring of women with active ED and past ED and offspring of age- and social class-matched controls. Lastly given that ED differ in terms of manifestation of specific behaviours, we explored the effect of maternal restrictive eating, purging (self-induced vomiting and laxative use for weight control) and binge eating on overall offspring methylation.

## Methods

### Sample

The Avon Longitudinal Study of Parents and Children (ALSPAC) is a longitudinal, population-based, extensive prospective study of women and their children, set up in the 1990s to investigate the effects of environment, genetic and other factors on child health and development [17, 18]. All pregnant women living in the geographical area of Avon, UK, who were expected to deliver their baby between 1 April 1991 and 31 December 1992 were invited to take part in the study. Uptake was high and those enrolled represented approximately 85% of the eligible population. ALSPAC recruited 14,541 pregnant women; all women gave informed and written consent. The study website contains details of all the data that is available through a fully searchable data dictionary: http://www.bris.ac.uk/alspac/researchers/data-access/data-dictionary/. Data are accessible to all bona fide researchers via the ALSPAC study. However, ALSPAC is required by its ethical approvals to manage access through an application process (which applied equally to the authors of this manuscript).

We selected women whose offspring had available DNA methylation data from cord blood (*n* = 1018) as part of the Accessible Resource for Integrated Epigenomic Studies (ARIES; for details, see http://www.ariesepigenomics.org.uk/), a representative sub-set of ALSPAC [18–20]. Offspring were not excluded from the study based on gestational age, prematurity, low birth weight and non-singleton pregnancies.

### Exposure (maternal ED)

Amongst women with offspring methylation data from cord blood (*n* = 1018), maternal active ED was determined via three sources: (a) questionnaires at 12 weeks enquiring about having had a recent ED; (b) questionnaires at 18 and 32 weeks in pregnancy on ED behaviours and cognitions [including specific questions about loss of control eating, purging (self-induced vomiting or laxative use for weight loss) and the weight and shape concern subscales of the Eating Disorder Examination Questionnaire (EDE-q), a well-validated instrument [21], as previously described in [21, 22]; and (c) validated semi-structured interviews recently carried out as part of a two-phase lifetime ED prevalence study [3].

Women were defined as having had an active ED in pregnancy (*n* = 21) if they (a) reported having had an active ED in pregnancy (AN or BN) (*n* = 11) or (b) reported symptoms consistent with an ED in pregnancy (loss of control eating, purging, high weight and shape concern in the context of underweight (*n* = 10)); or (c) reported symptoms consistent with an ED diagnosis in pregnancy on interviews (*n* = 7) (see Table 1). The majority (52.4%) of women were categorised as having active ED in pregnancy based on two out of three of the above sources.

**Table 1:** Sample characteristics

	Active ED (*n* = 21)	Past ED (*n* = 43)	Controls (*n* = 126)
Age, mean (SD)	26.4 (5.9)	29.1 (5.2)	28.4 (5.3)
BMI pre-pregnancy, mean (SD)	20.2 (4.4)	21.4 (2.5)	23.1 (3.9)
Smoking in pregnancy, *n* (%)	12 (57.1%)	9 (20.9%)	20 (15.9%)^a^
Child gender (female), *n* (%)	12 (57.1%)	22 (51.2%)	69 (54.8%)
Energy intake (kJ/day), mean (SD)	6769 (1969)	6714 (2239)	7474 (1950)

^a^One subject had missing data on smoking status

Women who self-reported having suffered from an ED prior to pregnancy, had no ED symptoms in pregnancy, and did not report any symptoms consistent with an ED in pregnancy were categorised as having suffered from a past ED (*n* = 43).

We also categorised women according to broad ED diagnostic type (restrictive, purging, binge eating, binge-purge) according to symptoms reported during and prior to pregnancy. Amongst women with active ED, 14 (66.7%) had a restrictive ED, 3 (14.3%) a binge-purge-type ED, 3 (14.3%) a purge-type ED and 1 (4.8%) a binge eating ED. Amongst women with past ED, 25 had a restrictive-type ED (58.1%), 10 (23.3%) a binge-purge-type ED and 4 (9.3%) a purge-type ED.

Two age- and social class-matched controls were selected for each case (*n* = 126).

### Socio-demographic data

Socio-demographic (maternal social class, maternal age) data, smoking in pregnancy, and weight and height pre-pregnancy were obtained by self-completion questionnaire at 12, 18 and 32 weeks’ gestation; body mass index (BMI) was calculated as pre-pregnant weight/height^2^. Total energy intake was collected via food frequency questionnaire (FFQ), completed by the women at 32 weeks’ gestation [10]. Offspring gender was obtained from obstetric records.

### Methylation measurements and quality control

DNA was extracted and was bisulfite converted using the Zymo EZ DNA MethylationTM kit (Zymo, Irvine, CA). After the conversion, DNA methylation level was measured using the Illumina Infinium® HumanMethylation450k BeadChip in line with standard protocols at the University of Bristol, UK.

The arrays were scanned using an Illumina iScan, and initial quality review was assessed using GenomeStudio (version 2011.1). Further quality checks included CpG detection *p* value test which removed the samples whose average detection *p* value is > 0.01. We also used several study specific variables and created multidimensional scaling (MDS) plots to identify any potential outliers, but all samples were within the thresholds. The measures of DNA methylation were computed as betas (*β*-value) using the minfi package in the R statistical programming language [23]. Methylation beta values range from 0 (no cytosine methylation) to 1 (completely cytosine methylated). Raw beta values were normalised using the functional normalisation method of the minfi package [24]. We excluded SNPs, probes with a detection *p* value > 0.05 in more than 5% samples and features on sex chromosomes. After excluding these features, 468,782 probes were left for analysis.

### Statistical analyses

We used multiple linear regression models to explore the effect of maternal ED on offspring cord blood methylation using the CpGassoc R package [25].

Firstly, cord blood in offspring of mothers with active ED, past ED and controls were compared. We profiled overall methylation levels amongst these three groups. We calculated a median beta value for each individual and then computed a median beta for each exposure group for comparisons. Crude and adjusted analyses were performed. Adjusted analyses included bisulfite conversion batch (BCD) to adjust for batch effects, and principal components (computed using principal component analysis (PCA) [26–28]. PCA was carried out to adjust for any unknown batch effects. Only those principal components (PC) were included which captured most of the variance and were not associated with the exposure. Maternal smoking status and child gender were included in all adjusted analyses as covariates. These covariates were used as fixed effect to minimise the computational burden.

Cell-type correction was applied using the reference-based Houseman method [29] in the minfi package in R. This method estimates the relative proportions of six white blood cell subtypes (CD4+ T lymphocytes, CD8+ T lymphocytes, NK (natural killer) cells, B lymphocytes, monocytes and granulocytes), based on a standard reference population [30]. Effect sizes and % methylation are reported.

We analysed four linear regression models including model 1: a crude regression of offspring DNA methylation on maternal ED group (as a predictor); model 2: as model 1 with adjustment for BCD and first six PCs (batches); model 3: as model 2 with additional adjustment for covariates (smoking and child gender); and model 4: as model 3 with additional adjustment for cell counts. We show results of Model 4, the fully adjusted model, throughout the paper. In EWAS results, we highlighted cross-hybridising probes as these probes can interfere with accurate detection of methylation levels [31].

For EWAS analyses, we computed the genomic inflation factor and generated quantile-quantile (QQ), Manhattan and volcano plots to compare the genome-wide distribution of *p* values and effect sizes across different analyses.

Methylation of two CpGs sites (uncorrected *p* value < 10^−7^) identified in fully adjusted EWAS analysis of active ED versus controls was profiled in the three exposure groups: controls, active ED, and past ED.

In order to determine whether any effects of active ED might be due to maternal underweight, we carried out sensitivity analyses to compare our top hits with those found in cord blood of offspring of underweight mothers in ALSPAC [16] and in a meta-analysis of sustained maternal smoking during pregnancy and cord blood methylation [32].

Linear regression analyses were performed to investigate the effects of specific ED type on offspring cord blood methylation after removing cross-hybridising probes (*n* = 440,643).

Post-hoc analyses investigated whether cord blood methylation differed in offspring of women with active vs. past restrictive-type ED.

## Results

Overall, 189 women (*n* = 21 with active ED, *n* = 43 with past ED, *n* = 125 healthy controls) had data on exposure, outcomes and relevant confounders under study. One healthy control was dropped from the study sample due to missing data on smoking in pregnancy.

Socio-demographic characteristics of the women under study are shown in Table 1. Women with current and past ED had lower average daily energy intake compared to controls (*F* = 2.8, *p* = 0.06). The majority of women in the active and past ED group had a restrictive-type ED.

### Whole-genome methylation

We removed cross-hybridising probes and calculated medians in order to plot and compare the overall methylation levels across the three groups (controls, active ED and past ED) (Additional file 1: Figure S1). Cord blood of offspring of women with active ED had the lowest methylation level (49.1%; 95% confidence intervals 50.5–47.7%); offspring of controls had the highest global methylation levels (52.4%; 95% CI 53.0–51.0%). The offspring of women with past ED had a lower global methylation level compared to those of controls (49.2%; 95% CI 50.7–47.7.0%) and slightly higher than the active ED group**.** An ANOVA showed medians were statistically different across groups (*p* = 0.008).

### Identification of differentially methylated CpGs

We carried out EWAS analyses to identify the association between maternal eating disorder and epigenome-wide DNA methylation in offspring cord blood measured using the Illumina HumanMethylation450 Beadchip. After adjustment for potential confounders and estimated white cell counts, differences were shown in two CpG sites between offspring of women with active ED and controls (all *p* < 10^−7^) (see Table 2) (Additional file 1: Figure S2 shows the relevant QQ and Manhattan plot), and in two CpGs between offspring of women with past ED and controls (all *p* < 10^−6^) (see Table 2). The top two CpGs identified in analysis of active ED and controls also passed Bonferroni correction (1.06 × 10^−7^ corrected for 468,782 associations). No significantly different CpGs were identified in offspring of mothers with active vs. past ED.

**Table 2:** Differentially methylated CpG sites in women with active ED (*n* = 21) and past ED (*n* = 43) and controls (*n* = 125)

CpG	Chromosome	Gene	Effect size	SE	*P* value	Bonferroni corrected	Cross-hybridising Probes
*Active ED* vs. *controls*							
cg21146184	22	near *ATXN10*	0.055	0.008448	1.60 × 10^−8^	0.007	No
cg10177197	1	*DHCR24*	−0.124	0.020012	6.94 × 10^−8^	0.032	No
*Past ED* vs. *controls*							
cg05963962	10	*LOC84856*	0.08165	0.01462	3.57 × 10^−7^	0.16	Yes
cg11081833	22	*LGALS2*	0.070354	0.012624	3.74 × 10^−7^	0.17	No

In particular, offspring of women with active ED had a hypomethylated region in cg10177197, mapping to the *DHCR24* gene, which corresponds to the gene encoding 3-β-hydroxysterol delta-24 reductase, an enzyme that catalyses the last step in the biosynthesis of cholesterol (also known as seladin 1). The second CpG site identified was cg21146184 on chromosome 22, which is near the *ATXN10* gene (the gene encoding Ataxin 10—a protein that may be involved in neuron survival, neuron differentiation and neuritogenesis) [32].

Two loci were differentially methylated in offspring of women with past ED vs. controls, and these trended for significance following Bonferroni correction: cg05963962, corresponding to *LOC84856 (LINC00839* a long intergenic non-protein coding element*)* on chromosome 10, and cg11081833, corresponding to a soluble beta-galactoside binding lectin (*LGALS2*) gene. This gene and its polymorphisms have been implicated in the metabolic syndrome [33], inflammation and immune response. Additional file 1: Figure S3 and Figure S4 shows methylation levels across groups in these CpGs.

In order to determine whether weight status partially explained our findings, we compared our EWAS top CpG sites (*p* value < 10^−4^) with results from a recent ALSPAC study on the effects of maternal over- and underweight and offspring cord blood methylation [16]. We considered the fully adjusted model reported in [16] in relation to maternal pre-pregnancy BMI (*n* = 284,972 probes) to compare the CpG sites identified in our analysis of active ED versus controls, and our top two hits were not present in [16]. The direction of effect was not consistent for the majority of the sites, the previously reported effect sizes were smaller, and *p* values were larger ranging from 0.01 to 0.99 (Additional file 2: Table S1). We also compared the CpG sites identified in our study in offspring of past ED versus controls with CPGs identified in [16], and the top CpG sites in our study were not found in the previously reported study [16]. In agreement with the previous comparison, the effect sizes in [16] were smaller and *p* values were larger ranging from 0.07 to 0.9 (Additional file 2: Table S2).

We compared the CpG sites identified in fully adjusted EWAS of active ED versus controls (*p* value < 10^−4^) with FDR significant sites found in a study of sustained maternal smoking in pregnancy (*n* = 6074 probes), and our top two hits were not present in [32]. There were only three overlapping CpGs (Additional file 2: Table S3). We also compared the CpG sites identified in our study in offspring of past ED versus controls with CPGs identified in [32] and four CpGs overlapped that include our second top site (Additional file 2: Table S4).

We investigated the association between ED diagnostic type (restrictive ED, purging ED, and binge eating and purging ED)—across active and past ED—and whole-genome methylation (results are shown in Table 3). In adjusted analyses, all ED diagnostic types predicted lower offspring cord blood methylation levels compared to controls: restrictive-type ED (*ß* = − 0.011, *p* = 0.15), purging-type ED (ß = − 0.026, *p* = 0.08), and binge + purge-type ED (− 0.009, *p* = 0.4) (see Table 3). Results were comparable when cross-hybridising probes were removed.

**Table 3:** Whole-genome cord blood methylation levels in offspring of women across ED types and controls: adjusted linear regression

ED type	*ß* (SE)	*p* value	*R* ^2^, *p* value
Restrictive ED (*n* = 39)	− 0.011 (0.008)	0.15	0.84, 2.7 × 10^−15^
Binge purge ED (*n* = 13)	− 0.009 (0.012)	0.42	
Purging ED (*n* = 7)	− 0.026 (0.015)	0.08	
Covariates:			
Smoking	0.01 (0.007)	0.14	
Gender	− 0.002 (0.006)	0.67	

Adjusted for maternal smoking in pregnancy, child gender, cell count and bisulfite conversion batch

### Post-hoc analyses

As the restrictive ED group was the largest, we were able to also explore differences in whole-genome methylation amongst women with active restrictive-type ED and past restrictive-type ED. Offspring of women with active restrictive-type ED had significantly lower average whole-genome methylation (*ß* = − 0.07 (95% CI: − 0.069, − 0.077), *p* = 0.01) compared to women with past restrictive-type ED in adjusted analyses.

## Discussion

This is the first study to investigate cord blood methylation in offspring born to mothers with eating disorders. As such, it provides novel and unique preliminary evidence of likely effects of maternal ED on global methylation and specific methylation signatures in domains relevant to metabolism and neuronal development. This study builds not only on existing evidence on effects of maternal ED on child development but also on novel findings highlighting the importance of genes relevant to metabolism in risk for ED [34, 35], and the first locus to be identified as being significantly associated with AN at genome-wide level [36].

Maternal eating disorders are associated with negative developmental outcomes across a range of behavioural and physical aspects. Pregnant women with active eating disorders also differ from healthy women in terms of their eating, diet and nutritional intakes and stress/anxiety levels [10, 11, 37–39]. Hence, we hypothesised that maternal eating disorders, especially if active during pregnancy, would be associated with differential methylation patterns in offspring cord blood. Offspring of mothers with active ED had in fact lower global methylation, by 3.3%, compared to offspring of age- and socio-demographic status-matched non-ED women. Interestingly, offspring of women with past ED had global methylation levels slightly higher than offspring of women with active ED, but nevertheless 3% lower than offspring of non-ED women. Children of women with restrictive-type ED and purge-type ED had the largest differences in global methylation compared to controls, although the number of women with purging behaviours only was small. In line with results indicating active pregnancy ED was associated with lowest global methylation levels, exploratory analyses indicated that restrictive eating in pregnancy was associated with lower offspring cord blood methylation compared to restrictive eating prior to pregnancy. We also identified intriguing significant differentially methylated CpGs in regions/genes relevant to metabolism, and neuronal development were associated with both maternal active and past ED.

Maternal diet and nutritional intake are amongst the major factors influencing the developing foetus [40]. Methylation depends on the availability of methyl group donors, mostly derived from diet. Therefore, hypomethylation has been shown to ensue diets with multiple methyl-related compounds nutrient deficiencies [41]. Epigenetic mechanisms have been suggested as potential mediating factors between maternal exposures (under and over-nutrition) and offspring adiposity. Evidence from the ALSPAC study also suggests that cord blood global hypomethylation is associated with maternal underweight and that hypomethylated sites [associated with maternal underweight] were negatively associated with offspring adiposity in later life. This suggests a possible mechanism for intergenerational foetal programming. Given the overlap between underweight and some ED (particularly restrictive ones), the fact that our top hypomethylated sites were not present amongst those associated with maternal underweight in ALSPAC strengthens our hypotheses that the effects seen in our study might not simply be secondary to maternal underweight. Cord blood hypomethylation has also been shown as a result of maternal pregnancy smoking [42] in humans. Although women with ED have higher prevalence of smoking in pregnancy [7, 22], smoking was adjusted for in all analyses. Corroboration that the effect of maternal ED on cord blood methylation might be driven by eating behaviours or their epiphenomena comes from our findings on ED-type showing lower methylation levels in offspring of mothers with restrictive- and purge-type ED (and within the restrictive ED subgroup, those women who had restrictive ED in pregnancy).

Cord blood methylation has also been suggested as a potential biomarker of altered child neurodevelopment [43], and research linking specific gene methylation and child neurodevelopmental outcomes has shown promising results. Our study highlights interesting differences in differentially methylated CpGs between offspring of ED mothers and controls. In particular, we identified differential methylation in genes relevant to lipid synthesis (*DHCR24*, *LGALS2*) [44]. *DHCR24* is responsive to lipid levels [45] and oestrogen [44], both likely to be abnormal in active ED and therefore possibly relevant to foetal programming. In fact, there is evidence that expression of *DHCR24* is relevant to foetal programming. *DHCR24* hepatic transcription is differentially induced by maternal undernutrition and low-protein diet in rats [46] and mice [47]; expression of *DHCR24* and related genes in the placenta was associated with foetal growth [48]. Two differentially methylated CpGs identified were in and near genes relevant to general pathways of normal embryonic and placental development (*LGALS2*) and neurogenesis *(ATXN10*)*.* These findings provide initial preliminary insights into possible biological effects of maternal ED; mapping onto existing evidence of both (a) developmental effects of maternal ED [4] and (b) risk pathways likely to be relevant to the intergenerational cycle of risk of ED [9].

Our findings have to be interpreted in the context of strengths and limitations. This is the first study to investigate methylation in offspring of women with ED. ED were ascertained using a triangulation method relying on self-report and interviews; we were able to test the effect of active ED vs. past ED. Given the nature of the sample, we were able to match controls to cases and adjust for confounders known to affect methylation levels, e.g. smoking. This study, however, is limited by the relatively small sample size, which nevertheless allowed exploration of the effect of ED type on the outcome. Other limitations include that no technical validation (e.g. pyrosequencing) was performed; studies have shown that the Illumina microarrays are less sensitive to extreme methylation levels [49, 50].

## Conclusions

This study provides for the first time evidence of the effect of maternal ED on early biological markers of offspring development, i.e. cord blood methylation. We showed important effects on genome-wide methylation levels and methylation of specific genes relevant to metabolism. Replication of our findings will be important. Recent studies have shown persistence of methylation levels into childhood [51]; hence, understanding whether differences identified in methylation are persistent and whether they are associated with adverse developmental outcomes will allow a more in-depth understanding of developmental risk pathways relevant to the intergenerational transmission of ED and broader psychopathology.

## Additional files

Additional file 1: Figure S1. Whole-genome cord blood methylation in offspring of active ED, past ED, and control women. **Figure S2.** QQ and Manhattan plots: association between ED and offspring cord blood DNA methylation^1^. **Figure S3.** Methylation levels of cg21146184 with *p* value < 10^−7^ identified in fully adjusted EWAS analysis of active ED versus controls. **Figure S4.** Methylation levels of cg10177197 with *p* value < 10^−7^ identified in fully adjusted EWAS analysis of active ED versus controls. (DOCX 398 kb)

Additional file 2: Table S1. Comparison of  top CpG sites (*p*-value < 10^−4^) found in EWAS of active ED versus controls with results from ALSPAC study of maternal pre-pregnancy BMI. **Table S2.** Comparison of  top CpG sites (*p*-value < 10^−4^) found in EWAS of past ED versus controls with results from ALSPAC study of maternal pre-pregnancy BMI. **Table S3.** Comparison of  top CpG sites (*p*-value < 10^−4^) found in EWAS of active ED versus controls with results of sustained maternal smoking in pregnancy. **Table S4.** Comparison of top CpG sites (*p*-value < 10^−4^) found in EWAS of past ED versus controls with results of sustained maternal smoking in pregnancy. (DOCX 49.2 kb)

## Abbreviations

ALSPAC: Avon longitudinal study of parents and children
AN: Anorexia nervosa
BCD: Bisulfite conversion batch
BED: Binge eating disorder
BMI: Body mass index
BN: Bulimia nervosa
ED: Eating disorder
EWAS: Epigenome-wide association study
PC: Principal components
