# Supplementary material for: Maternal eating disorders affect offspring cord blood DNA methylation: a prospective study
Source: Clin Epigenetics. 2017 Oct 27;9:120. doi: 10.1186/s13148-017-0418-3 (PMC5659017; doi:10.1186/s13148-017-0418-3)
Supplement: Supplementary file 1 — Whole-genome cord blood methylation in offspring of active ED, past ED, and control women. Figure S2. QQ and Manhattan plots: association between ED and offspring cord blood DNA methylation1. Figure S3. Methylation levels of cg21146184 with p value < 10−7 identified in fully adjusted EWAS analysis of active ED versus controls. Figure S4. Methylation levels of cg10177197 with p value < 10−7 identified in fully adjusted EWAS analysis of active ED versus controls. (DOCX 398 kb) [file 13148_2017_418_MOESM1_ESM.docx]

**Figure S1: Whole genome cord blood methylation in offspring of active ED, past ED and control women**

**
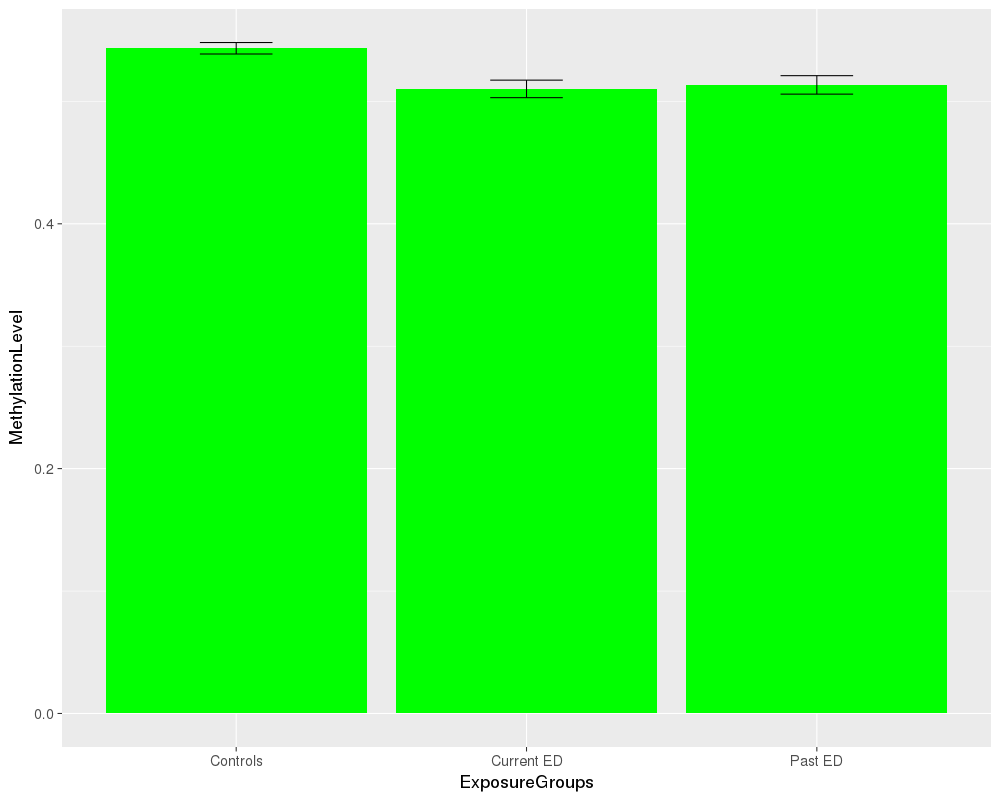
**

**Figure S2: QQ and Manhattan plots: association between ED and offspring cord blood DNA methylation^1^**


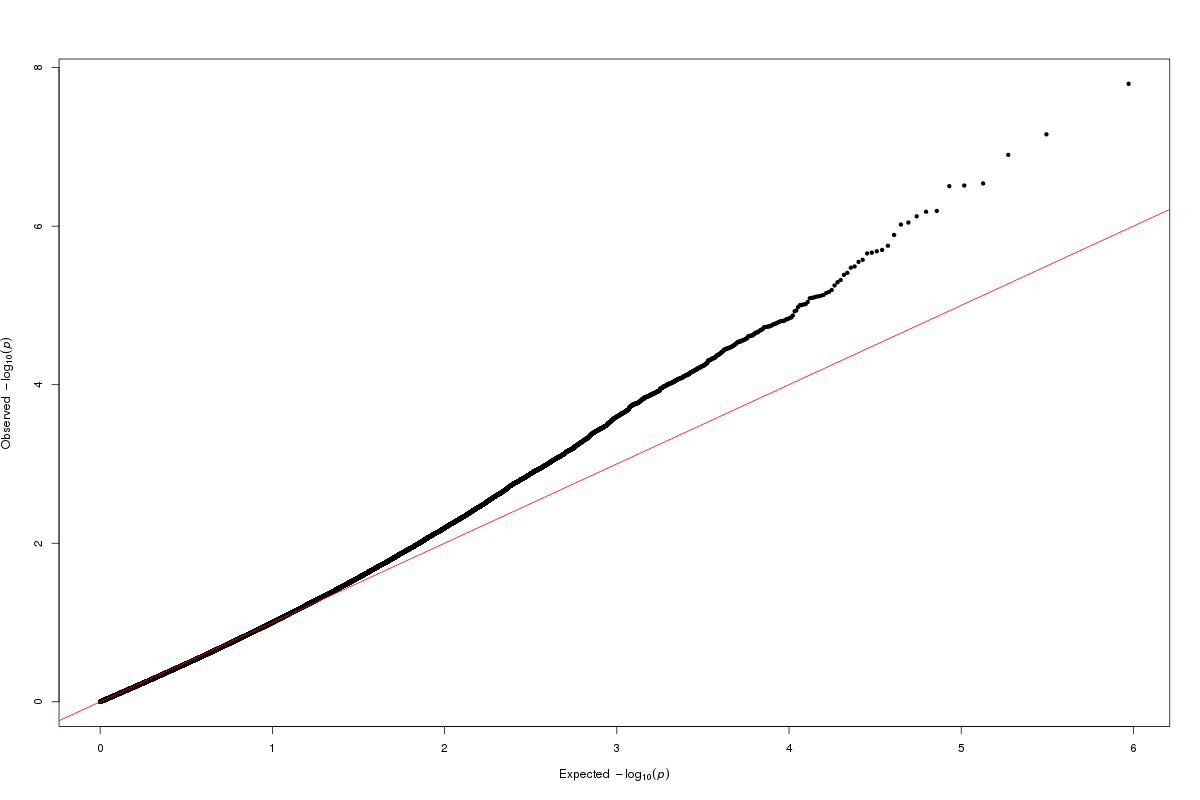


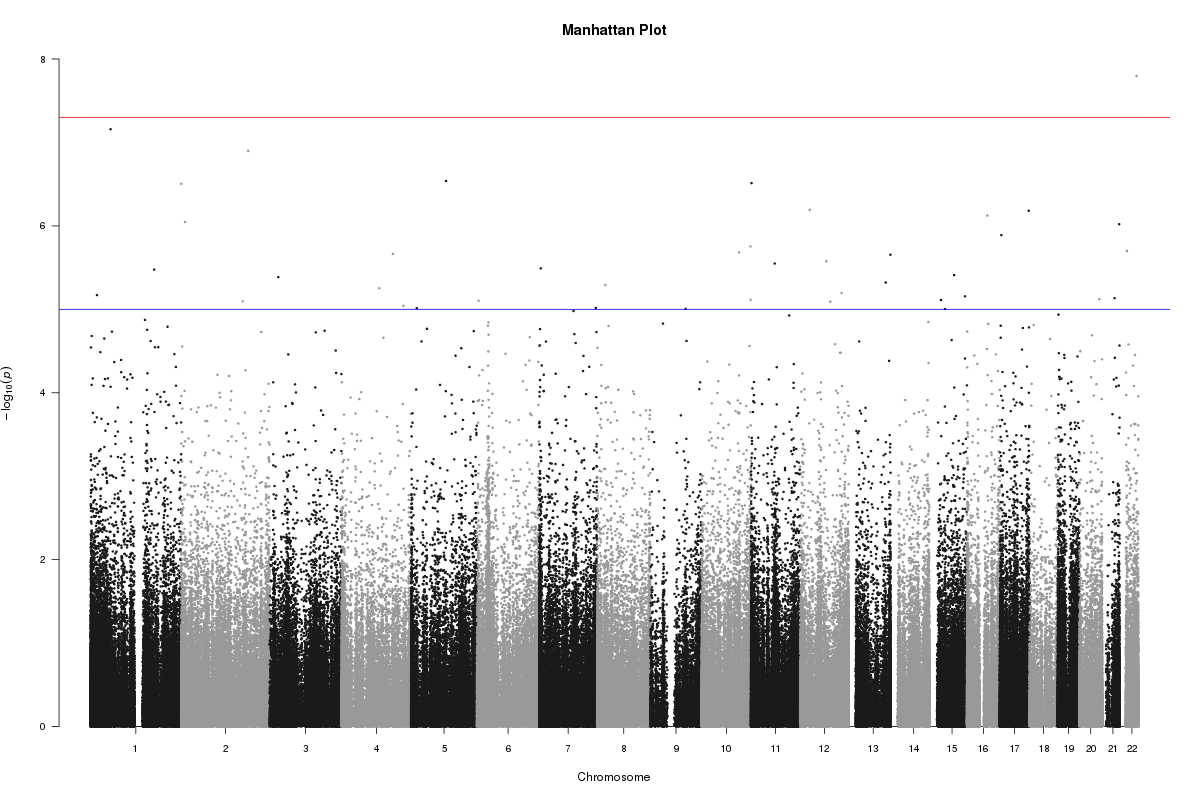


1:The model was adjusted for covariates and estimated cell counts. The uncorrected –log10(P-values) are plotted.

**Legend: Active ED (red), past ED (light blue), and controls (green)**

**Figure S3: Methylation levels of cg21146184 with p-value < 10^-7^ identified in fully adjusted EWAS analysis of Active ED versus controls**


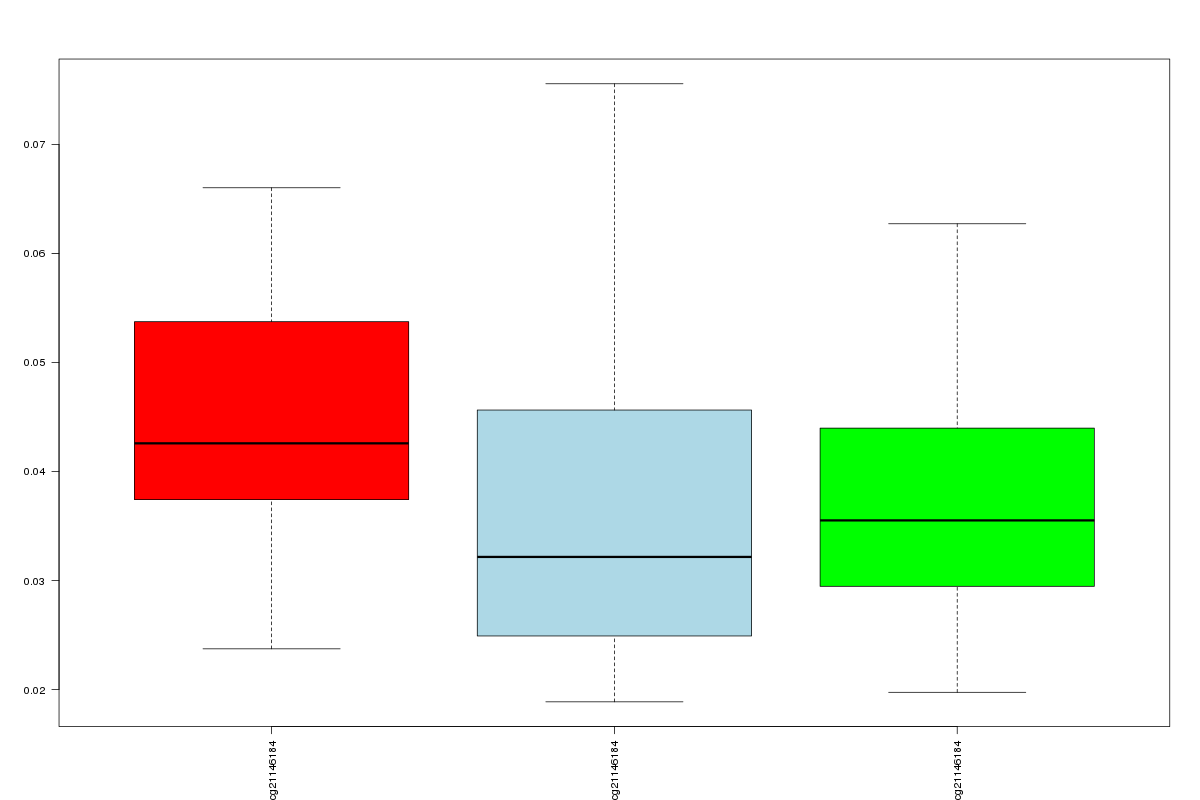


**Legend: Active ED (red), past ED (light blue), and controls (green)**

**Figure S4: Methylation levels of cg10177197 with p-value < 10^-7^ identified in fully adjusted EWAS analysis of Active ED versus controls**

**
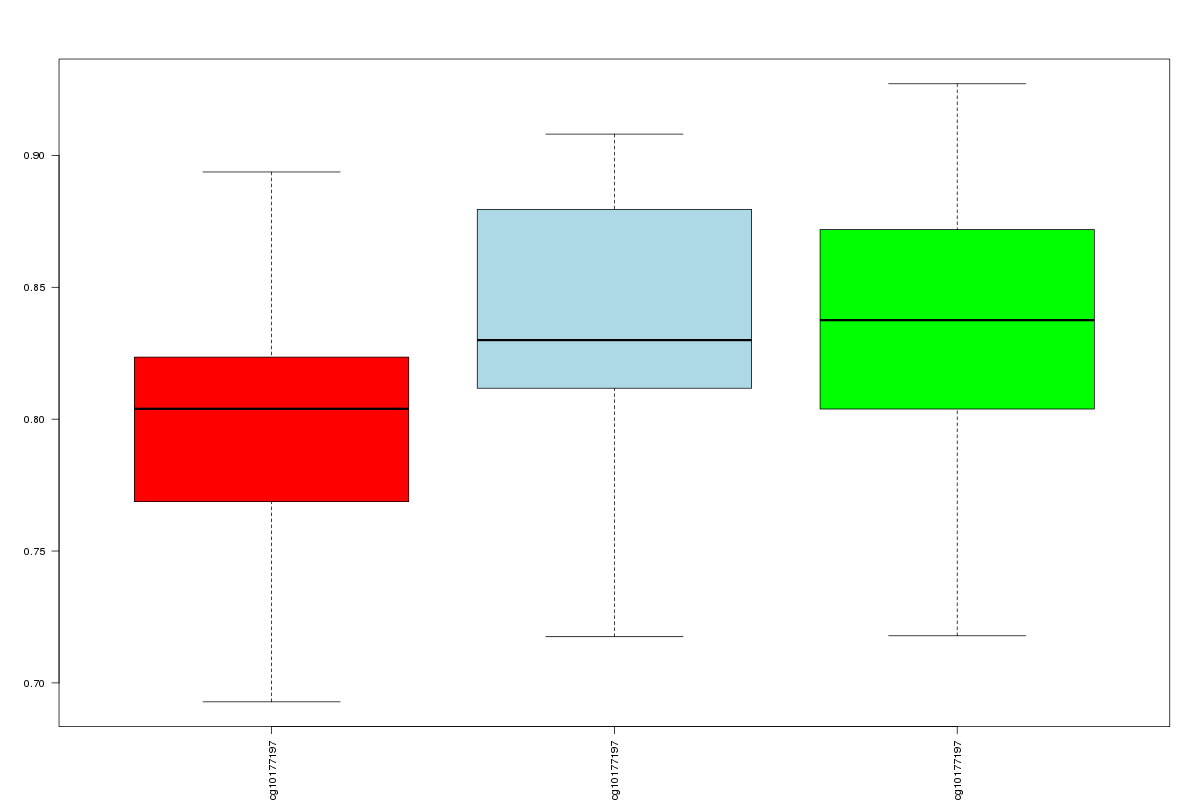
**

**Legend: Active ED (red), past ED (light blue), and controls (green)**
