# Supplementary material for: Maternal eating disorders affect offspring cord blood DNA methylation: a prospective study
Source: Clin Epigenetics. 2017 Oct 27;9:120. doi: 10.1186/s13148-017-0418-3 (PMC5659017; doi:10.1186/s13148-017-0418-3)
Supplement: Supplementary file 2 — Comparison of top CpG sites (p-value < 10−4) found in EWAS of active ED versus controls with results from ALSPAC study of maternal pre-pregnancy BMI. Table S2. Comparison of top CpG sites (p-value < 10−4) found in EWAS of past ED versus controls with results from ALSPAC study of maternal pre-pregnancy BMI. Table S3. Comparison of top CpG sites (p-value < 10−4) found in EWAS of active ED versus controls with results of sustained maternal smoking in pregnancy. Table S4. Comparison of top CpG sites (p-value < 10−4) found in EWAS of past ED versus controls with results of sustained maternal smoking in pregnancy. (DOCX 49.2 kb) [file 13148_2017_418_MOESM2_ESM.docx]

**Table S1:** Comparison of  top CpG sites (p-value <10^-4^) found in EWAS of active ED versus controls with results from ALSPAC study of maternal pre-pregnancy BMI.

| **CpG** | **Gene** | **CHR** | **Position** | **Effect-ED** | **P-value-ED** | **SE-ED** | **Effect-BMI** | **P-value-BMI** | **SE-BMI** |
| --- | --- | --- | --- | --- | --- | --- | --- | --- | --- |
| cg15779295 |  | 2 | 184125352 | -0.20139 | 1.26E-07 | 0.03337 | 0.002382 | 0.207343 | 0.001887 |
| cg08856347 | FAM150B | 2 | 289390 | 0.013688 | 3.13E-07 | 0.002363 | -2.82E-06 | 0.982128 | 0.000126 |
| cg05568797 | BHLHE41 | 12 | 26275050 | 0.011212 | 6.41E-07 | 0.002002 | -1.01E-05 | 0.848461 | 5.27E-05 |
| cg04337928 |  | 17 | 79320473 | -0.02865 | 6.57E-07 | 0.005122 | 0.000144 | 0.026748 | 6.47E-05 |
| cg02884524 | ANKFY1 | 17 | 4075375 | -0.07047 | 1.29E-06 | 0.013021 | 0.000349 | 0.288934 | 0.000328 |
| cg03965795 |  | 10 | 102431021 | -0.02675 | 2.07E-06 | 0.005063 | -0.0001 | 0.574309 | 0.00018 |
| cg00716604 | TBC1D15 | 12 | 72233552 | -0.0037 | 2.66E-06 | 0.000709 | 6.16E-05 | 0.546931 | 0.000102 |
| cg02344238 | FRMD8 | 11 | 65153777 | 0.050966 | 2.82E-06 | 0.009804 | -0.00011 | 0.326276 | 0.00011 |
| cg27658048 | SDK1 | 7 | 4201594 | -0.19941 | 3.22E-06 | 0.03863 | 9.14E-05 | 0.90296 | 0.000749 |
| cg04858586 | RABGAP1L | 1 | 174843971 | 0.092134 | 3.34E-06 | 0.017881 | -0.00017 | 0.635557 | 0.000368 |
| cg18980373 |  | 15 | 67145803 | 0.074456 | 3.88E-06 | 0.014568 | 0.000269 | 0.476957 | 0.000378 |
| cg09023624 |  | 3 | 23784916 | 0.142804 | 4.11E-06 | 0.028028 | -0.00206 | 0.081365 | 0.00118 |
| cg13244417 | TMTC4 | 13 | 101327186 | 0.029226 | 4.77E-06 | 0.005783 | 1.08E-06 | 0.982062 | 4.79E-05 |
| cg07830103 | NFKB1 | 4 | 103422050 | 0.024701 | 5.59E-06 | 0.00493 | 5.68E-05 | 0.500736 | 8.43E-05 |
| cg11586330 | LHX5 | 12 | 113900751 | 0.070932 | 6.38E-06 | 0.014263 | 1.54E-06 | 0.993702 | 0.000195 |
| cg05380570 | ARHGEF10L | 1 | 17866686 | 0.057564 | 6.75E-06 | 0.011611 | 2.24E-05 | 0.881748 | 0.000151 |
| cg19087643 | INPP5A | 10 | 134503483 | -0.07441 | 7.66E-06 | 0.015116 | 0.000244 | 0.521896 | 0.000381 |
| cg18599162 | MTMR15 | 15 | 31217417 | -0.0918 | 7.74E-06 | 0.018661 | 0.001527 | 0.01552 | 0.000629 |
| cg22659356 | STK39 | 2 | 169104591 | 0.080972 | 8.03E-06 | 0.016493 | 9.83E-05 | 0.454224 | 0.000131 |
| cg25054754 | PALLD | 4 | 169754328 | 0.027635 | 9.04E-06 | 0.005667 | -0.00024 | 0.34869 | 0.000254 |
| cg14667871 | FBXL7 | 5 | 15500833 | 0.00429 | 9.70E-06 | 0.000883 | -2.84E-05 | 0.728534 | 8.19E-05 |
| cg22894805 | MGA | 15 | 41983773 | 0.135988 | 9.92E-06 | 0.028038 | 0.000825 | 0.253907 | 0.000722 |
| cg21853542 | SGCE | 7 | 94286559 | 0.161786 | 1.05E-05 | 0.03346 | 0.001082 | 0.359206 | 0.001179 |
| cg27587645 | BTBD2 | 19 | 2015311 | 0.029644 | 1.16E-05 | 0.006167 | -3.27E-05 | 0.444321 | 4.28E-05 |
| cg07572052 | HIST2H2AA4 | 1 | 149821168 | 0.227697 | 1.34E-05 | 0.047774 | -0.00027 | 0.660377 | 0.000606 |
| cg09985481 | CDC42BPB | 14 | 103411899 | -0.12613 | 1.42E-05 | 0.026556 | 0.000434 | 0.292208 | 0.000412 |
| cg10524192 | SIGMAR1 | 9 | 34637478 | 0.004783 | 1.48E-05 | 0.00101 | -0.00011 | 0.186296 | 8.04E-05 |
| cg20059140 | GNAL | 18 | 11751516 | 0.038243 | 1.54E-05 | 0.008092 | -0.00015 | 0.557896 | 0.00026 |
| cg21745320 | SMG6 | 17 | 2207495 | 0.059613 | 1.57E-05 | 0.012628 | -0.00019 | 0.155865 | 0.000133 |
| cg04773818 | NRG1 | 8 | 31496966 | 0.054749 | 1.58E-05 | 0.011604 | -0.00024 | 0.130933 | 0.000161 |
| cg16262415 | NEK2 | 1 | 211848604 | 0.018411 | 1.61E-05 | 0.003906 | 3.81E-05 | 0.383007 | 4.36E-05 |
| cg08983961 | NPB | 17 | 79859947 | 0.078394 | 1.65E-05 | 0.016654 | -0.00018 | 0.623435 | 0.000358 |
| cg17413194 | GNA13 | 17 | 63053996 | -0.05953 | 1.67E-05 | 0.012659 | 0.000411 | 0.465405 | 0.000563 |
| cg08052882 | NNT | 5 | 43602666 | 0.028471 | 1.71E-05 | 0.006062 | 3.79E-05 | 0.583598 | 6.91E-05 |
| cg01813334 | EIF3B | 7 | 2394150 | 0.041952 | 1.72E-05 | 0.008937 | 3.22E-05 | 0.696115 | 8.24E-05 |
| cg24517925 | MSTO2P | 1 | 155715448 | 0.024038 | 1.76E-05 | 0.005127 | -8.28E-05 | 0.098243 | 5.00E-05 |
| cg23351724 | IGSF10 | 3 | 151177604 | 0.08777 | 1.80E-05 | 0.018747 | -0.00046 | 0.526479 | 0.000718 |
| cg26524306 | NEURL1B | 5 | 172113857 | -0.07119 | 1.83E-05 | 0.015218 | -0.00105 | 0.057813 | 0.00055 |
| cg23890044 | RAB40C | 16 | 640595 | -0.01902 | 1.84E-05 | 0.004068 | -3.56E-05 | 0.494874 | 5.21E-05 |
| cg23676961 | OMA1 | 1 | 59012576 | 0.020325 | 1.85E-05 | 0.004348 | 7.61E-05 | 0.211258 | 6.08E-05 |
| cg01697972 | ANKZF1 | 2 | 220093858 | 0.015518 | 1.87E-05 | 0.003322 | 2.31E-05 | 0.548217 | 3.84E-05 |
| cg07048143 | DNAJB6 | 7 | 157199479 | -0.13318 | 1.87E-05 | 0.028516 | 0.00021 | 0.711002 | 0.000567 |
| cg01138164 |  | 7 | 96648447 | 0.114115 | 1.98E-05 | 0.024516 | 1.50E-05 | 0.96024 | 0.000301 |
| cg27306802 | MEGF6 | 1 | 3510561 | 0.194995 | 2.09E-05 | 0.042025 | -0.00044 | 0.376945 | 0.000497 |
| cg23160829 |  | 6 | 145219031 | -0.10596 | 2.16E-05 | 0.022882 | 0.000335 | 0.428647 | 0.000423 |
| cg27025247 | SMG6 | 17 | 2207482 | 0.072068 | 2.19E-05 | 0.015577 | -0.00041 | 0.354023 | 0.000441 |
| cg13448605 | RSPO1 | 1 | 38100467 | 0.0246 | 2.24E-05 | 0.005325 | 2.17E-05 | 0.827567 | 9.94E-05 |
| cg27019126 | RAX | 18 | 56940693 | 0.018698 | 2.26E-05 | 0.00405 | -0.00021 | 0.165359 | 0.000149 |
| cg25224094 | FOXB1 | 15 | 60296273 | 0.030389 | 2.34E-05 | 0.006595 | -2.90E-05 | 0.756533 | 9.36E-05 |
| cg17475200 |  | 9 | 99482469 | 0.076949 | 2.39E-05 | 0.016725 | -0.00012 | 0.236022 | 0.000105 |
| cg21413947 |  | 1 | 165329957 | -0.11843 | 2.40E-05 | 0.025745 | 0.000339 | 0.585023 | 0.00062 |
| cg06137852 |  | 13 | 28545283 | -0.04154 | 2.42E-05 | 0.009035 | 3.63E-05 | 0.758441 | 0.000118 |
| cg16925459 | HDAC9 | 7 | 18548581 | 0.147641 | 2.43E-05 | 0.032122 | 0.001431 | 0.273242 | 0.001305 |
| cg18453884 | SNRPF | 12 | 96252338 | 0.081155 | 2.61E-05 | 0.017737 | 1.93E-05 | 0.815309 | 8.25E-05 |
| cg04951739 | ZNF70 | 22 | 24093724 | 0.022618 | 2.64E-05 | 0.004946 | 3.23E-05 | 0.653443 | 7.20E-05 |
| cg09758896 | EBF3 | 10 | 131694575 | -0.11686 | 2.74E-05 | 0.025616 | -0.00103 | 0.39421 | 0.00121 |
| cg06527438 |  | 6 | 170546536 | -0.08525 | 2.76E-05 | 0.018695 | 0.001061 | 0.459708 | 0.001434 |
| cg08910524 | C1orf27 | 1 | 186347746 | 0.096278 | 2.84E-05 | 0.021153 | 0.000277 | 0.682194 | 0.000676 |
| cg22371227 | FAM5B | 1 | 177139876 | 0.097422 | 2.85E-05 | 0.021406 | 2.47E-05 | 0.833469 | 0.000118 |
| cg01006034 | CCNL2 | 1 | 1322789 | -0.11477 | 2.86E-05 | 0.025227 | 0.001943 | 0.23195 | 0.001624 |
| cg13614962 |  | 8 | 962418 | 0.082748 | 2.89E-05 | 0.018198 | -0.00137 | 0.445415 | 0.001792 |
| cg01632097 | HSPA9 | 5 | 137897226 | -0.07832 | 2.93E-05 | 0.017239 | 0.000322 | 0.321775 | 0.000325 |
| cg06259881 | IRX3 | 16 | 54321165 | 0.050217 | 2.99E-05 | 0.011068 | -0.00025 | 0.130843 | 0.000163 |
| cg22920300 | SOX2OT | 3 | 181441364 | 0.091112 | 3.12E-05 | 0.020135 | -0.00064 | 0.143545 | 0.000439 |
| cg14037036 | PIGV | 1 | 27113573 | 0.01232 | 3.26E-05 | 0.00273 | -0.00012 | 0.047298 | 6.10E-05 |
| cg08141615 | MGC14436 | 12 | 110172214 | -0.05994 | 3.31E-05 | 0.013298 | -2.73E-05 | 0.78257 | 9.88E-05 |
| cg10264012 |  | 6 | 78359965 | -0.04925 | 3.41E-05 | 0.010946 | -6.94E-05 | 0.571203 | 0.000122 |
| cg20967887 | PGBD5 | 1 | 230459114 | -0.12604 | 3.44E-05 | 0.02803 | 0.000866 | 0.322731 | 0.000876 |
| cg10221896 |  | 16 | 79263595 | -0.20752 | 3.46E-05 | 0.046165 | -1.84E-05 | 0.983675 | 0.000897 |
| cg03037622 | RBM15B | 3 | 51430415 | -0.06959 | 3.47E-05 | 0.015485 | -9.31E-05 | 0.738717 | 0.000279 |
| cg20112838 | UNC13A | 19 | 17797656 | 0.026917 | 3.51E-05 | 0.005994 | 0.000111 | 0.417705 | 0.000136 |
| cg14106119 | NHP2L1 | 22 | 42084222 | 0.002869 | 3.52E-05 | 0.000639 | -3.83E-05 | 0.483725 | 5.46E-05 |
| cg27469991 | IFNGR2 | 21 | 34776637 | 0.108152 | 3.80E-05 | 0.024207 | -7.76E-05 | 0.634876 | 0.000163 |
| cg07683276 | C6orf94 | 6 | 144184358 | 0.108438 | 4.10E-05 | 0.024392 | 0.000233 | 0.787563 | 0.000866 |
| cg04895321 | NMT2 | 10 | 15210264 | -0.00321 | 4.22E-05 | 0.000723 | -2.67E-05 | 0.477941 | 3.77E-05 |
| cg22128605 |  | 6 | 168402503 | -0.08201 | 4.27E-05 | 0.018495 | -0.00018 | 0.729942 | 0.000508 |
| cg01002030 | RAVER2 | 1 | 65211677 | 0.055033 | 4.29E-05 | 0.012415 | -0.00015 | 0.385579 | 0.000171 |
| cg18329187 | CKB | 14 | 103989711 | 0.053083 | 4.41E-05 | 0.011996 | -0.00035 | 0.462298 | 0.000478 |
| cg19120389 |  | 11 | 117686162 | 0.025201 | 4.54E-05 | 0.005706 | 3.43E-05 | 0.460553 | 4.64E-05 |
| cg06545189 | ADK | 10 | 75936595 | 0.002804 | 4.60E-05 | 0.000636 | -2.55E-06 | 0.933552 | 3.06E-05 |
| cg05062333 | EYA4 | 6 | 133562269 | 0.024232 | 4.62E-05 | 0.005493 | -8.45E-05 | 0.265727 | 7.59E-05 |
| cg14343334 | UBE2O | 17 | 74387045 | -0.13378 | 4.85E-05 | 0.030427 | -0.00014 | 0.799422 | 0.000542 |
| ch.7.2865377R | | 7 | 137692325 | -0.03385 | 4.89E-05 | 0.007702 | 6.44E-05 | 0.360546 | 7.03E-05 |
| cg12657297 |  | 1 | 234667363 | -0.176 | 4.90E-05 | 0.040055 | 0.001472 | 0.107682 | 0.000914 |
| cg02543268 | ATP10B | 5 | 160201261 | -0.08484 | 4.91E-05 | 0.01931 | 0.000484 | 0.47223 | 0.000673 |
| cg25597797 | GIPC3 | 19 | 3589762 | -0.11656 | 5.31E-05 | 0.026667 | -0.00062 | 0.566352 | 0.001072 |
| cg10753836 | HOXD9 | 2 | 176989485 | 0.125615 | 5.38E-05 | 0.028764 | -0.00078 | 0.151366 | 0.00054 |
| cg07964754 | COTL1 | 16 | 84650202 | -0.1657 | 5.48E-05 | 0.03799 | -0.00135 | 0.435746 | 0.001726 |
| cg06458092 | TSPAN12 | 7 | 120497559 | 0.009467 | 5.48E-05 | 0.002171 | 2.31E-05 | 0.666183 | 5.36E-05 |
| cg24699433 | EIF5A | 17 | 7210249 | -0.02917 | 5.64E-05 | 0.006702 | -3.14E-05 | 0.54518 | 5.18E-05 |
| cg18116902 |  | 1 | 84326666 | 0.092065 | 5.66E-05 | 0.021156 | -0.00021 | 0.221439 | 0.00017 |
| cg01836687 | psiTPTE22 | 22 | 17081987 | 0.078427 | 5.71E-05 | 0.018032 | 0.000245 | 0.418505 | 0.000303 |
| cg14761246 | MCF2L2 | 3 | 182968758 | -0.12962 | 5.79E-05 | 0.02983 | 0.000759 | 0.157209 | 0.000536 |
| cg13950948 | BCAN | 1 | 156627399 | 0.035576 | 5.84E-05 | 0.008192 | -0.00017 | 0.117656 | 0.000107 |
| cg16543923 | LOC220729 | 3 | 197353980 | 0.048948 | 5.97E-05 | 0.011288 | 0.000116 | 0.279102 | 0.000107 |
| cg18078387 | SCIN | 7 | 12610124 | 0.097389 | 6.08E-05 | 0.022485 | -0.00015 | 0.713539 | 0.000413 |
| cg25411534 | TSGA10 | 2 | 99758462 | 0.018539 | 6.08E-05 | 0.004281 | 4.47E-05 | 0.39839 | 5.28E-05 |
| cg24593272 |  | 10 | 102977637 | 0.062567 | 6.19E-05 | 0.014464 | -0.00015 | 0.334542 | 0.000155 |
| cg06638787 |  | 6 | 5997243 | 0.013633 | 6.19E-05 | 0.003152 | -5.74E-05 | 0.420444 | 7.12E-05 |
| cg23296369 | SHD | 19 | 4290672 | -0.09992 | 6.42E-05 | 0.023158 | 0.001151 | 0.105901 | 0.000711 |
| cg11592634 | SH3GL1 | 19 | 4370001 | 0.309387 | 6.49E-05 | 0.071755 | 0.000441 | 0.583049 | 0.000804 |
| cg11412935 | GFI1 | 1 | 92950086 | 0.010968 | 6.50E-05 | 0.002544 | -0.00011 | 0.32503 | 0.000113 |
| cg25141894 | DENND2C | 1 | 115164619 | 0.130604 | 6.60E-05 | 0.030325 | 0.001557 | 0.075717 | 0.000875 |
| cg17383940 |  | 21 | 38936403 | -0.01612 | 6.63E-05 | 0.003744 | -4.21E-05 | 0.510968 | 6.40E-05 |
| cg03383250 |  | 1 | 47972688 | -0.05194 | 6.71E-05 | 0.012073 | -5.44E-05 | 0.868566 | 0.000329 |
| cg27056555 | ICMT | 1 | 6293422 | -0.05709 | 6.71E-05 | 0.013271 | 0.000577 | 0.121056 | 0.000372 |
| cg02636814 | KIAA0754 | 1 | 39881324 | -0.07333 | 6.85E-05 | 0.017067 | 0.000152 | 0.791908 | 0.000578 |
| cg24544798 |  | 11 | 48989769 | 0.098683 | 6.91E-05 | 0.022985 | -0.00062 | 0.549935 | 0.001034 |
| cg06279276 | B3GNT9 | 16 | 67184164 | 0.023139 | 7.23E-05 | 0.005406 | 2.56E-05 | 0.874213 | 0.000162 |
| cg27335720 | ZNF529 | 19 | 37064375 | 0.007968 | 7.40E-05 | 0.001865 | -8.51E-05 | 0.346136 | 9.03E-05 |
| cg03486010 | TUB | 11 | 8102311 | 0.024981 | 7.43E-05 | 0.005847 | -0.00015 | 0.021305 | 6.63E-05 |
| cg21220264 | PIGG | 4 | 520093 | -0.1439 | 7.48E-05 | 0.033698 | 6.68E-05 | 0.925076 | 0.00071 |
| cg03243700 | WDR5 | 9 | 137000940 | 0.017361 | 7.48E-05 | 0.004066 | -2.83E-05 | 0.614643 | 5.63E-05 |
| cg00985104 | TMEM111 | 3 | 10028380 | 0.008527 | 7.49E-05 | 0.001997 | 4.03E-05 | 0.16049 | 2.86E-05 |
| cg10904028 | LRP1 | 12 | 57590920 | -0.127 | 7.53E-05 | 0.029753 | -0.00039 | 0.531216 | 0.00062 |
| cg00760203 | PLXDC1 | 17 | 37254921 | 0.10112 | 7.70E-05 | 0.023729 | 0.000252 | 0.39521 | 0.000296 |
| cg22353755 |  | 19 | 28397061 | 0.118033 | 7.72E-05 | 0.027702 | 0.000257 | 0.802585 | 0.001027 |
| cg04213089 | L3MBTL4 | 18 | 6284382 | -0.18652 | 7.83E-05 | 0.043818 | 0.000541 | 0.796486 | 0.002097 |
| cg10581281 | MITF | 3 | 69788259 | 0.031506 | 7.91E-05 | 0.007407 | -0.00016 | 0.03618 | 7.85E-05 |
| cg17447867 |  | 20 | 47016568 | -0.05374 | 7.92E-05 | 0.012635 | 0.000593 | 0.263215 | 0.00053 |
| cg21961270 | PRDM16 | 1 | 3307177 | 0.074121 | 8.05E-05 | 0.017446 | -7.67E-05 | 0.822838 | 0.000342 |
| cg14482322 | TARSL2 | 15 | 102264062 | 0.079778 | 8.15E-05 | 0.018794 | 7.52E-05 | 0.475598 | 0.000105 |
| cg06068897 |  | 21 | 45577667 | 0.110664 | 8.20E-05 | 0.026081 | -8.17E-05 | 0.665464 | 0.000189 |
| cg08903465 |  | 1 | 234917213 | 0.066643 | 8.22E-05 | 0.015709 | -0.00079 | 0.073135 | 0.000442 |
| cg11598005 | FDFT1 | 8 | 11665962 | 0.038149 | 8.24E-05 | 0.008994 | -0.00013 | 0.366133 | 0.000143 |
| cg08534256 | DNAL4 | 22 | 39189722 | 0.021479 | 8.30E-05 | 0.005066 | -1.50E-05 | 0.852597 | 8.07E-05 |
| cg24113973 |  | 12 | 114889042 | 0.051238 | 8.31E-05 | 0.012087 | -6.80E-05 | 0.618936 | 0.000137 |
| cg21982941 | HS3ST3B1 | 17 | 14213447 | -0.18728 | 8.34E-05 | 0.04419 | 0.001133 | 0.320741 | 0.00114 |
| cg15248353 | DYRK1A | 21 | 38738865 | 0.005913 | 8.41E-05 | 0.001396 | -0.00011 | 0.013412 | 4.47E-05 |
| cg11811705 |  | 1 | 55462689 | 0.007364 | 8.47E-05 | 0.00174 | -0.00019 | 0.195575 | 0.00015 |
| cg09557342 | DCTN6 | 8 | 30014267 | 0.035741 | 8.50E-05 | 0.008445 | -7.35E-05 | 0.17157 | 5.37E-05 |
| cg14690980 | HGF | 7 | 81400745 | 0.067167 | 8.55E-05 | 0.015876 | -0.00064 | 0.366827 | 0.000706 |
| cg13431165 | ROPN1B | 3 | 125691192 | 0.045981 | 8.63E-05 | 0.010875 | -4.46E-05 | 0.330074 | 4.58E-05 |
| cg22589511 | SMAD3 | 15 | 67458522 | -0.11135 | 8.66E-05 | 0.026344 | 0.000559 | 0.28218 | 0.00052 |
| cg15820062 |  | 8 | 23584404 | 0.016976 | 8.79E-05 | 0.004021 | 3.43E-05 | 0.778922 | 0.000122 |
| cg23715559 | NUP98 | 11 | 3819094 | 0.014223 | 8.86E-05 | 0.00337 | -6.69E-05 | 0.128856 | 4.40E-05 |
| cg18270280 | HIAT1 | 1 | 100504403 | 0.012078 | 8.90E-05 | 0.002863 | -0.00011 | 0.037549 | 5.42E-05 |
| cg13732164 |  | 9 | 134669414 | -0.10331 | 9.04E-05 | 0.024514 | -2.24E-06 | 0.997887 | 0.000846 |
| cg08818829 | DNAH5 | 5 | 13810279 | 0.253365 | 9.14E-05 | 0.060168 | -0.00076 | 0.632504 | 0.001582 |
| cg23782820 |  | 8 | 58130467 | -0.14 | 9.19E-05 | 0.033261 | -0.00168 | 0.152401 | 0.00117 |
| cg04818943 | LOC100130015 | 16 | 90114200 | 0.018085 | 9.22E-05 | 0.004298 | 9.41E-05 | 0.573493 | 0.000167 |
| cg20981058 | ZNF585A | 19 | 37663744 | 0.018874 | 9.41E-05 | 0.004492 | -9.11E-05 | 0.182121 | 6.82E-05 |
| cg17109042 | ANKS1B | 12 | 99139387 | 0.084873 | 9.44E-05 | 0.020202 | -0.00029 | 0.073284 | 0.000162 |
| cg20728105 | SCIN | 7 | 12610689 | 0.11111 | 9.54E-05 | 0.026467 | -3.02E-05 | 0.92989 | 0.000343 |
| cg03852144 | GLRX | 5 | 95159639 | 0.111211 | 9.56E-05 | 0.026494 | -9.38E-05 | 0.831004 | 0.000439 |
| cg14639897 | MATN2 | 8 | 98881124 | 0.025747 | 9.60E-05 | 0.006136 | -7.73E-05 | 0.210226 | 6.16E-05 |
| cg22594309 | SYT2 | 1 | 202679504 | 0.010325 | 9.76E-05 | 0.002463 | -3.77E-05 | 0.641811 | 8.10E-05 |
| cg11331739 |  | 4 | 53734538 | -0.0812 | 9.81E-05 | 0.019381 | 0.00024 | 0.647427 | 0.000524 |
| cg24701267 |  | 3 | 72368071 | -0.11763 | 9.89E-05 | 0.028092 | 0.000261 | 0.741041 | 0.000789 |
|  |  |  |  |  |  |  |  |  |  |

**Table S2:** Comparison of  top CpG sites (p-value <10^-4^) found in EWAS of past ED versus controls with results from ALSPAC study of maternal pre-pregnancy BMI.

| **CpG** | **Gene** | **CHR** | **Position** | **Effect-ED** | **P-value-ED** | **SE-ED** | **Effect-BMI** | **P-value-BMI** | **SE-BMI** |
| --- | --- | --- | --- | --- | --- | --- | --- | --- | --- |
| cg11081833 | LGALS2 | 22 | 37975941 | 0.070354 | 3.74E-07 | 0.012624 | -0.00053 | 0.625854 | 0.001091 |
| cg16178491 | SLC5A8 | 12 | 101603996 | 0.040211 | 4.02E-06 | 0.008082 | 0.000271 | 0.080113 | 0.000155 |
| cg11861730 | ETS1 | 11 | 128392683 | 0.008428 | 8.13E-06 | 0.001759 | 5.62E-05 | 0.298778 | 5.41E-05 |
| cg00187889 | TAGAP | 6 | 159463103 | -0.07062 | 9.15E-06 | 0.01483 | 0.000444 | 0.670206 | 0.001043 |
| cg26834192 | AGPAT4 | 6 | 161561031 | -0.07698 | 9.77E-06 | 0.016225 | 0.001423 | 0.139849 | 0.000963 |
| cg10671054 | SCNN1A | 12 | 6456430 | 0.060019 | 1.24E-05 | 0.01282 | -0.00152 | 0.072177 | 0.000843 |
| cg04840494 | SERINC5 | 5 | 79550959 | 0.0308 | 1.79E-05 | 0.006719 | -5.74E-05 | 0.576318 | 0.000103 |
| cg03560090 | RBM42 | 19 | 36119752 | -0.00797 | 1.87E-05 | 0.001742 | -2.94E-05 | 0.507721 | 4.43E-05 |
| cg15288779 | RGMA | 15 | 93587978 | -0.1068 | 2.14E-05 | 0.023551 | -0.00071 | 0.554532 | 0.001194 |
| cg01193690 | | 13 | 28491615 | 28491615 | 2.46E-05 | 0.001007 | 0.000119 | 0.128565 | 7.85E-05 |
| cg09726240 | SHANK2 | 11 | 70672878 | 0.076697 | 2.48E-05 | 0.017062 | -0.00029 | 0.234624 | 0.000243 |
| cg15238694 | LOC144742 | 12 | 119740840 | 0.09687 | 2.93E-05 | 0.021764 | -0.00046 | 0.780369 | 0.001641 |
| cg02416336 | KRTAP6-2 | 21 | 31971025 | -0.04309 | 3.05E-05 | 0.009705 | -0.00044 | 0.480297 | 0.000618 |
| cg05705964 | CTPS | 1 | 41453012 | 0.058685 | 3.11E-05 | 0.013234 | -0.00048 | 0.542165 | 0.000792 |
| cg13763339 | SHANK2 | 11 | 70516627 | -0.03622 | 3.24E-05 | 0.00819 | -0.00032 | 0.504907 | 0.000476 |
| cg05460716 | DUSP27 | 1 | 167063879 | -0.03809 | 3.41E-05 | 0.008639 | -0.00051 | 0.147177 | 0.000349 |
| cg09595050 | PRDM8 | 4 | 81110205 | -0.12312 | 3.45E-05 | 0.02794 | 0.000424 | 0.71984 | 0.001183 |
| cg01907194 | TRMT61A | 14 | 104003370 | -0.06938 | 3.49E-05 | 0.015758 | -0.00095 | 0.313388 | 0.000945 |
| cg25743719 | HPCAL1 | 2 | 10443138 | 0.005751 | 3.83E-05 | 0.001314 | 3.34E-05 | 0.761022 | 0.00011 |
| cg18941614 | UBTF | 17 | 42287715 | 0.037953 | 4.11E-05 | 0.008708 | -3.98E-05 | 0.729298 | 0.000115 |
| cg07488381 | | 2 | 67235436 | 67235436 | 5.71E-05 | 0.009787 | -0.00016 | 0.707673 | 0.000439 |
| cg04872717 | | 3 | 197226107 | 197226107 | 5.84E-05 | 0.021271 | 0.00059 | 0.81758 | 0.002556 |
| cg19431241 | KCNK15 | 20 | 43374350 | 0.012222 | 6.28E-05 | 0.002882 | 2.47E-05 | 0.75324 | 7.84E-05 |
| cg02812767 | LOXL1 | 15 | 74218468 | 0.061787 | 6.99E-05 | 0.014673 | -0.00013 | 0.93728 | 0.001646 |
| cg01882880 | HOXB2 | 17 | 46623819 | 0.049184 | 7.19E-05 | 0.011702 | 0.000306 | 0.646765 | 0.000668 |
| cg15835825 | HTR5A | 7 | 154862030 | 0.008376 | 7.39E-05 | 0.001997 | -0.0001 | 0.085871 | 5.95E-05 |
| cg03664800 | LOXL2 | 8 | 23261312 | 0.00382 | 7.71E-05 | 0.000913 | 1.26E-05 | 0.771336 | 4.35E-05 |
| cg20398560 | | 2 | 168401330 | 168401330 | 7.71E-05 | 0.019954 | 0.001014 | 0.410529 | 0.001232 |
| cg00008488 | | 5 | 175199915 | 175199915 | 8.76E-05 | 0.007273 | -0.00013 | 0.315281 | 0.00013 |
| cg03801429 | SLC7A5 | 16 | 87894565 | -0.04161 | 8.90E-05 | 0.010044 | 0.000817 | 0.594445 | 0.001534 |

**Table S3:** Comparison of  top CpG sites (p-value <10^-4^) found in EWAS of active ED versus controls with results of sustained maternal smoking in pregnancy

| **CpG** | **Gene** | **CHR** | **Position** | **Effect-ED** | **P-value-ED** | **SE-ED** | **Effect-Smoking** | **P-value-Smoking** | **SE-Smoking** |
| --- | --- | --- | --- | --- | --- | --- | --- | --- | --- |
| cg04858586 | RABGAP1L | 1 | 174843971 | 0.092134 | 3.34E-06 | 0.017881 | -0.006 | 7.41E-08 | 0.001 |
| cg19087643 | INPP5A | 10 | 134503483 | -0.07441 | 7.66E-06 | 0.015116 | -0.005 | 5.90E-06 | 0.001 |
| cg22500876 | | 7 | 45187902 | -0.17046 | 5.90E-05 | 0.03928 | 0.007 | 2.20E-08 | 0.001 |

**Table S4:** Comparison of top CpG sites (p-value <10^-4^) found in EWAS of past ED versus controls with results of sustained maternal smoking in pregnancy

| **CpG** | **Gene** | **CHR** | **Position** | **Effect-ED** | **P-value-ED** | **SE-ED** | **Effect-Smoking** | **P-value-Smoking** | **SE-Smoking** |
| --- | --- | --- | --- | --- | --- | --- | --- | --- | --- |
| cg00187889 | TAGAP | 6 | 159463103 | -0.07062 | 9.15E-06 | 0.01483 | 0.004 | 0.00276 | 0.001 |
| cg15238694 | LOC144742 | 12 | 119740840 | 0.09687 | 2.93E-05 | 0.021764 | -0.004 | 3.96E-05 | 0.001 |
| cg09595050 | PRDM8 | 4 | 81110205 | -0.12312 | 3.45E-05 | 0.02794 | 0.004 | 0.000121 | 0.001 |
| cg02812767 | LOXL1 | 15 | 74218468 | 0.061787 | 6.99E-05 | 0.014673 | 0.006 | 9.52E-05 | 0.002 |
